# Neurocysticercosis—More Than a Neglected Disease

**DOI:** 10.1371/journal.pntd.0001964

**Published:** 2013-04-25

**Authors:** Theodore E. Nash, Siddhartha Mahanty, Hector H. Garcia

**Affiliations:** 1 Laboratory of Parasitic Diseases, National Institutes of Allergy and Infectious Diseases, National Institutes of Health, Bethesda, Maryland, United States of America; 2 Department of Microbiology, School of Sciences, and Center for Global Health - Tumbes, Universidad Peruana Cayetano Heredia, Lima, Perú; 3 Cysticercosis Unit, Instituto Nacional de Ciencias Neurologicas, Lima, Perú

Neurocysticercosis (NCC) is the most common cause of adult-acquired epilepsy worldwide and one the most frequent parasitic infections associated with chronic morbidity in the United States. Despite its importance [1]–[4], worldwide morbidity due to NCC is underappreciated and research is underfunded, and therefore researchers are unable to capitalize on recent advances that hold great promise to prevent millions of cases of epilepsy and to effectively treat viable brain infections.

Similar to most neglected tropical diseases, assessing the global burden of disease for NCC is very imprecise, and is particularly burdensome because of the insensitivity of serology as a diagnostic tool and the need for brain imaging to establish presence of infection and/or disease. These limitations are particularly evident in Africa, where infrastructure and availability of CT and/or MRI facilities are limited. NCC is common in many countries of Central and South America, Haiti, India, most of Africa, Southeast Asia, and parts of China. Estimates of the burden of cysticercosis and NCC, based upon detailed studies of endemic communities, were recently published [2], and the burden of disease is substantial [3]. Some communities showed a prevalence of greater than 35% for infection with cysticercosis by serology and brain imaging, and intracerebral calcifications indicative of NCC in 10%–20% of randomly studied individuals. Studies of persons with epilepsy in well-characterized communities found that 29% of epilepsy in endemic communities is attributable to NCC [5], which in some communities amounted to 1% or greater of the population. By conservative estimates, greater than 5 million cases of epilepsy worldwide, which are all preventable, are caused by NCC.

The life cycle of the pork tapeworm *Taenia solium* is complicated [1], [6]. The tapeworm lives in the human small intestine and sheds multiple proglottids, each containing 30,000–50,000 infectious ova. Proglottids may retain ova as they are excreted in feces, or liberate ova in the feces or around the anus. When free-roaming pigs ingest human stools with infectious ova, the liberated oncospheres penetrate the intestinal mucosa and are carried to the muscles, brain, and other tissues of the pig and establish as cysts which, when ingested by humans in poorly cooked pork, develop into tapeworms (taeniasis). Humans become infected with cysts (cysticercosis) following the accidental ingestion of ova-contaminated hands, food, or water. Because the life cycle of the parasite is complex and exacting, it is particularly susceptible to interruption, and simple interventions such as corralling pigs, preventing indiscriminate defecation, and fully cooking pork can potentially prevent human infection.

Advancements in our knowledge of the importance of NCC, the means to establish the diagnosis, and the re-purposing and use of drugs to treat disease has been phenomenal. It is easy to forget that a few decades ago the diagnosis of NCC was made infrequently, medical treatments were unavailable, and its role as a primary cause of adult onset epilepsy unknown. Despite these advances, the study of NCC lags behind most other major infections of the tropics, and further progress requires improved and more effective approaches as well as an increased and balanced commitment of resources.

The study of NCC is inherently difficult. The life cycle is near impossible to maintain in the laboratory, and for practical purposes most basic research cannot be performed in developed, non-endemic countries. The high cost of the logistics to establish and maintain the stages of the life cycle required for basic experiments is compounded by the difficult challenge of obtaining financial support for a neglected disease. Researchers have naturally turned to imperfect models of cestode infections that are employed to understand immune host response and host–parasite interactions, to develop in vitro and in vivo assays to understand how effective drugs work, and to discover safer and better drugs for use in humans. Controlled human treatment trials and observational studies are difficult to conduct because of their cost, need for frequent imaging, and requirement for long periods of follow up. As a consequence, a relatively small cadre of persons and laboratories are devoted to understanding the parasite and its disease manifestations.

Despite these impediments, there have been a number of notable advances in recent years that could lead to better treatments and control of disease. As examples, a potentially useful avenue of research has been the development of highly effective vaccines to the invading oncosphere larva in pigs, which are based on high levels of immunity that normally develops to the migrating larva [7]. Strategic use of vaccines could potentially prevent infection in the obligate intermediate host, eliminating taeniasis and NCC. Sensitive methods to diagnose tapeworm infections have been developed and are likely to play an important role in control of infection. Recombinant and/or synthesized antigens have been identified to replace *T. solium*–harvested antigens in antibody detection assays [8]. The *T. solium* genome, which has recently been published [9], offers an expanded armamentarium of tools to interrogate the biology of the parasite, identify molecules involved in host–parasite interactions, and discover more effective and safer cysticidal drugs. Complicated subarachnoid infection previously carried a dismal prognosis, which has now improved, most likely because of prolonged anthelmintic therapy, use of corticosteroids and other anti-inflammatory agents, and careful long-term assessments and use of brain imaging. Use of ventricular endoscopy to remove accessible ventricular cysts seems to result in decreased morbidity.

Other advances in the management of NCC still await further development. The treatment of NCC has improved considerably but is still suboptimal. The preponderance of evidence, based upon an excellent randomized blinded placebo controlled trial and earlier less rigorously controlled trials, showed that anthelminthic treatment of parenchymal NCC resulted in a decrease in generalized seizures during the subsequent 1–2 years. Unfortunately, the efficacy of the available cysticidal drugs, albendazole and praziquantel, is suboptimal, with cure rates between 40% and 50% at the usually recommended dosing. More effective and safer alternatives are needed. The natural history and prognosis of single enhancing degenerating lesions has been well defined. NCC is a chronic inflammatory infection of the brain and much of morbidity is directly or indirectly caused by inflammation. Corticosteroids have traditionally been used to control parasite-induced inflammation. How to use corticosteroids effectively is being studied and preliminary results indicate that rational use leads to decreased seizures [10]. Most persons with epilepsy due to NCC only have calcified granulomas that, in many cases, are the foci of seizures; half of such lesions develop sporadic episodes of perilesional edema around the calcifications [11]. The evidence thus far suggests the later seizures are brought about by intermittent inflammatory responses to the calcified granuloma, which, if confirmed, suggests a potential role for anti-inflammatory or immunomodulatory agents in the control of epilepsy due to NCC. Assays that detect parasite antigens (Ag) in serum and cerebrospinal fluid have been applied to more easily diagnose, characterize, and monitor active infections. Although generally proven predictive and helpful, the assays' sensitivity and specificity are still being defined in the different types of disease. These assays may be particularly helpful to determine effectiveness of treatment for subarachnoid disease and assist in deciding when to stop antihelminthic treatment. Nevertheless, treatments are prolonged and some patients suffer from serious morbidities such as strokes and corticosteroid side effects. Better drugs and treatment regimens will require the development of easy to use in vitro and in vivo drug screening methods [12].

A number of intervention strategies to control infection have also been realized. The introduction of oxfendazole as a single dose therapy for porcine cysticercosis, coproantigen detection for improved diagnosis of taeniasis, and as already mentioned, a highly efficacious TSOL18 pig vaccine, have made control of transmission a potentially achievable goal. Other vaccines also look promising. A series of attempts at control of human infections using mass chemotherapy with praziquantel or niclosamide as well as many interventions at the same time have been performed in several Latin American countries, and a large-scale elimination effort is already ongoing in northern Peru.

For the most part, the neuroscience community has not fully taken advantage of NCC as a natural model for research. It is the only common infectious disease or process where normal individuals are exposed en masse to a seizure-inducing agent. Questions such as how epilepsy develops, how inflammatory foci develop into epilepsy, and why some people develop epilepsy and some do not can be studied in this population. NCC is a chronic inflammatory condition of the brain that can be used to answer basic questions concerning the nature of brain inflammation and how best to control it.

Although progress has been made in research on NCC, there are a number of opportunities for studies that would lead to a better understanding of the pathobiology of NCC and its elimination.

1. Determine the extent and burden of disease worldwide. NCC is likely a common cause of serious morbidity worldwide and one of the most common morbid parasitic diseases in the United States.
2. Understand that great strides can be made with relatively few resources, for example:
  - Development of methods to test for new and effective drugs.
  - Use of existing, licensed immunomodulators to control treatment-induced inflammation and damaging inflammation in parenchymal and subarachnoid disease.
  - Effective use of vaccination in pigs.
3. Boost support for the relatively few programs devoted to NCC.
4. In the United States, realize that health care and research is hindered because those with disease have the least access to health care; they are commonly undocumented Central and South American immigrants. Often, the most minimal care and testing is allowed or given.

We call upon the tropical disease community and national and international funding institutions to enhance the support of programs and studies aimed at eliminating infection and disease and devising rational and more effective treatment (Table 1). In this instance, a small investment will change the lives of many, and may take us a long way towards elimination of a serious public health burden.

**Table 1:** Major goals and needs for the management and control of neurocysticercosis.

Goal	Needs
1. Understand the extent of the burden of infection of disease and define regions and populations that would benefit from more effective control measures	• Better ways to diagnose infection and/or organized methods to determine infection and disease. Need for imaging is limiting and expensive
2. Safer and better ways to treat viable parenchymal neurocysticercosis	• Better and safer regimens• New drugs, perhaps using analyses of the *T. solium* genome to define susceptible pathways• Methods for testing new drugs
3. How best to treat complicated subarachnoid disease	• Better drugs, as above• Measures of efficacy• Testing and use of safer long-term anti-inflammatory and immunomodulating drugs
4. Better and more effective ways to control seizures and epilepsy related to calcification	• Define mechanisms of seizures induced by calcifications• Evaluate whether immune modulators such as anti-inflammatory cytokine antibodies or immunosuppressive drugs can be applied to control seizures in NCC
5. Improve control strategies and make them available and affordable	• Make tools commercially available, at affordable prices, in cysticercosis-endemic regions• Effective methods to use vaccines to control infection in pigs, including new delivery mechanisms
